# Blood pressure control and components of the metabolic syndrome: the GOOD survey

**DOI:** 10.1186/1475-2840-8-51

**Published:** 2009-09-15

**Authors:** Walter Zidek, Lisa Naditch-Brûlé, Stefano Perlini, Csaba Farsang, Sverre E Kjeldsen

**Affiliations:** 1 Medizinische Klinik IV (Endocrinology and Nephrology), Charité, Berlin, Germany; 2 Sanofi Aventis, Paris, France; 3 Department of Internal Medicine, Fondazione IRCCS San Matteo, Università di Pavia, Pavia, Italy; 4 The Cardiometabolic Centre, St Imre Hospital, Budapest, Hungary; 5 Department of Cardiology, Ullevaal Hospital, Norway

## Abstract

**Background:**

The GOOD (Global Cardiometabolic Risk Profile in Patients with Hypertension Disease) survey showed that blood pressure control was significantly worse in hypertensive patients with metabolic syndrome and/or diabetes mellitus than in those with essential hypertension only. This analysis aimed to investigate which components of the metabolic syndrome are primarily associated with poor blood pressure control.

**Methods:**

The GOOD survey was designed as an observational cross-sectional survey in 12 European countries to assess the cardiometabolic risk profile in patients with essential hypertension. Investigators were randomly selected from a list of general practitioners (70% of investigators) and a list of specialists such as internists, cardiologists and hypertension specialists (30% of investigators). Data from 3,280 outpatients with hypertension, aged at least 30 years who were receiving antihypertensive treatment or had newly diagnosed hypertension according to the European Society of Hypertension and the European Society of Cardiology criteria, were included in the analyses. Blood pressure control, body mass index (BMI), waist circumference, serum triglycerides, total and high density lipoprotein (HDL) cholesterol measurements were compared in patients with diabetes mellitus and metabolic syndrome, with diabetes mellitus only, with metabolic syndrome only, and with neither metabolic syndrome nor diabetes mellitus.

**Results:**

The highest blood pressure values were found in patients with metabolic syndrome with or without diabetes mellitus. Blood pressure was significantly lower in patients with diabetes mellitus only. The highest BMI, waist circumference and serum triglycerides, and the lowest HDL cholesterol levels among the groups studied occurred in patients with metabolic syndrome, either with or without diabetes mellitus.

**Conclusion:**

Among the components of the metabolic syndrome, it is not impaired glucose tolerance which is associated with the poor response to antihypertensive treatment. Instead, visceral obesity and dyslipidemia components of the metabolic syndrome, i.e. hypertriglyceridemia and low HDL cholesterol levels, are associated with resistance to antihypertensive treatment.

## Background

In a previous paper, in which the principal results of the Global Cardiometabolic Risk Profile in Patients with Hypertension Disease (GOOD) survey were reported, the main finding was that blood pressure control was significantly worse in hypertensive patients with diabetes mellitus and/or metabolic syndrome [1]. The prevalence of metabolic syndrome and type 2 diabetes was significantly higher in patients with uncontrolled blood pressure compared with those with controlled blood pressure. Ninety five percent of patients with both metabolic syndrome and type 2 diabetes had uncontrolled blood pressure. Diabetes mellitus and metabolic syndrome present with partly overlapping features. Of note, patients with metabolic syndrome without diabetes mellitus were associated with a similarly poor blood pressure control as those with diabetes mellitus. The components of the metabolic syndrome, besides hypertension, are impaired glucose tolerance, dyslipidemia and obesity, especially central obesity, as measured by waist circumference. Therefore, in view of the principal results of the GOOD survey, we aimed to investigate which components of the metabolic syndrome are primarily associated with poor blood pressure control.

## Methods

The GOOD survey was designed as an observational cross-sectional survey in 12 European countries to assess the cardiometabolic risk profile in patients with essential hypertension [1]. The study was conducted in Belgium, Germany, Hungary, Italy, the Netherlands, Norway, Portugal, Slovenia, Spain, Sweden, Turkey and the United Kingdom between 6 October, 2006 and 16 May, 2007. Investigators were randomly selected from a list of general practitioners (70% of investigators) and a list of specialists such as internists, cardiologists and hypertension specialists (30% of investigators). The lists contained 3- to 10-fold the number of investigators needed.

The first patient to be seen by physicians each working day who fulfilled the inclusion criteria were asked to participate. In the case of decline the next patient was asked to participate. Maximally two patients per day were recruited. Further details of the selection procedure have been published earlier [1].

Patients included in the survey were outpatients with hypertension, aged at least 30 years, who were receiving antihypertensive treatment or had newly diagnosed hypertension according to the European Society of Hypertension and European Society of Cardiology (ESH/ESC) criteria [2,3], which was confirmed on the day of inclusion in the study.

Exclusion criteria were pregnancy, menstruation, hospitalization, secondary hypertension, fever, known renal disease (serum creatinine > 177 μmol/l) and current drug treatment and/or concomitant conditions that could affect microalbuminuria measurements.

### Data collection

The following data were collected during the patients' visits: weight, height, waist circumference, seated blood pressure (two measurements taken after at least 3 minutes' rest), heart rate at rest, urinary albumin excretion, cardiometabolic risk factors, including duration of hypertension, history of diabetes, cardiovascular disease or stroke, lifestyle factors including alcohol consumption, physical exercise and smoking habits, and laboratory measurements of fasting blood glucose, fasting lipid profile and serum creatinine. Furthermore, data on current antihypertensive medications, other cardiovascular, antithrombotic, lipid, glucose and uric acid-lowering drugs were collected. Patients with incomplete records were not excluded. Metabolic syndrome was diagnosed according to the National Cholesterol Education Program Adult Treatment Panel III (ATP III) criteria [4], i.e. at least three of the following had to be present: blood pressure ≥ 130/85 mmHg; waist circumference ≥ 102 cm (men) or ≥ 88 cm (women); triglycerides ≥ 1.69 mmol/l or on drug treatment for elevated triglycerides; HDL cholesterol < 1.03 mmol/l (men) or < 1.29 mmol/l (women) or on drug treatment for reduced HDL cholesterol; fasting glucose ≥ 5.55 mmol/l or on drug treatment for elevated glucose. In this study, history or treatment of hypertension were not counted as criteria for metabolic syndrome as they applied to all patients.

The study was conducted in accordance with the principles laid down by the 18th World Medical Assembly (Helsinki, 1964) and all subsequent amendments. The study was also conducted in accordance with the European guidelines for Good Epidemiology Practice (GEP), and Proper Conduct in Epidemiologic Research (European Federation, IEA and "societies", 2004). Each participating country ensured that all necessary regulatory submissions (e.g., IRB/IEC) were performed in accordance with local regulations including local data protection regulations.

### Statistics

Among the hypertensive patients studied, four groups were compared: patients with metabolic syndrome and diabetes mellitus, with metabolic syndrome but no diabetes mellitus, with diabetes mellitus but no metabolic syndrome, and with neither diabetes mellitus nor metabolic syndrome. The haemodynamic, clinical and metabolic parameters were tested for significance using analysis of variance with Tukey's test as a post-hoc test.

## Results

**Figure 1:**
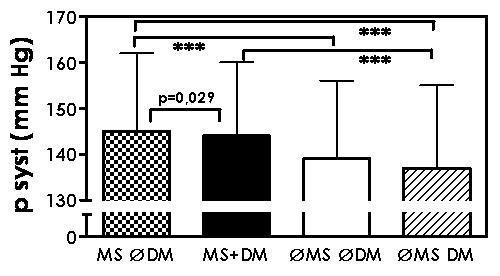
Systolic blood pressure (mean ± SD) in patients with metabolic syndrome, but no diabetes mellitus (MS ∅DM), with metabolic syndrome and diabetes mellitus (MS+DM), with neither metabolic syndrome nor diabetes mellitus (∅MS ∅DM), and with diabetes mellitus only (∅MS DM); *** p < 0.001.

In total, 3,464 outpatients were recruited in the survey and of these 3,280 were included in these analyses. The regional distribution of the patients has been described earlier [2]. Figure 1 and Table 1 show systolic blood pressure in hypertensive patients with metabolic syndrome (*n *= 1033), with diabetes mellitus (*n *= 206), with both (*n *= 864), and with none of these (*n *= 1177). Systolic blood pressure was highest in patients with metabolic syndrome with or without diabetes mellitus. Furthermore, systolic blood pressure was significantly higher in patients with metabolic syndrome only, than in patients with diabetes mellitus only.

**Table 1:** Systolic and diastolic blood pressure (P sys, P dia), body mass index (BMI), waist circumference and serum lipids (mean ± SD) in the different groups studied.

	**MS ∅DM**	**MS + DM**	**∅MS ∅DM**	**∅MS DM**
P sys (mm Hg)	145 ± 17	144 ± 16	139 ± 17	137 ± 18

P dia (mm Hg)	87 ± 10	83 ± 9	83 ± 10	80 ± 10

BMI (kg/m^2^)	30,7 ± 4,6	31,7 ± 4,9	27,2 ± 4,1	27,6 ± 4,4

Waist circumference (cm)	108,2 ± 11,5	110,5 ± 13,2	98,2 ± 10,0	97,6 ± 9,8

Total cholesterol (mmol/l)	5,52 ± 0,03	5,17 ± 0,04	5,33 ± 0,03	4,87 ± 0,08

Triglycerides (mmol/l)	2,12 ± 0,03	2,25 ± 0,03	1,29 ± 0,03	1,24 ± 0,06

HDL cholesterol (mmol/l)	1,25 ± 0,01	1,20 ± 0,01	1,55 ± 0,01	1,47 ± 0,03

**Figure 2:**
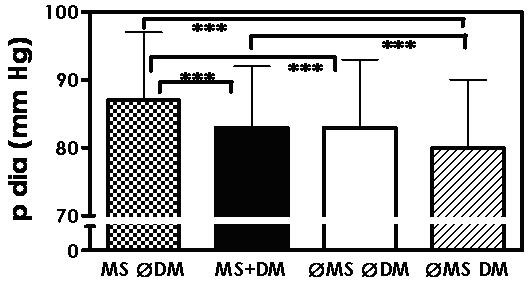
Diastolic blood pressure (mean ± SD) in patients with metabolic syndrome, but no diabetes mellitus (MS ∅DM), with metabolic syndrome and diabetes mellitus (MS+DM), with neither metabolic syndrome nor diabetes mellitus (∅MS ∅DM), and with diabetes mellitus only (∅MS DM); *** p < 0.001.

**Figure 3:**
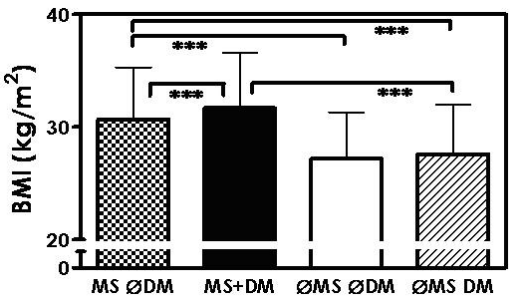
BMI (mean ± SD) in patients with metabolic syndrome, but no diabetes mellitus (MS ∅DM), with metabolic syndrome and diabetes mellitus (MS+DM), with neither metabolic syndrome nor diabetes mellitus (∅MS ∅DM), and with diabetes mellitus only (∅MS DM); *** p < 0.001.

**Figure 4:**
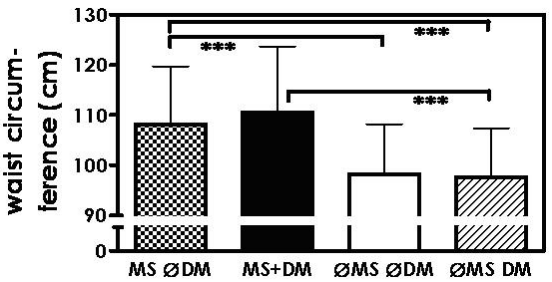
Waist circumference (mean ± SD) in patients with metabolic syndrome, but no diabetes mellitus (MS ∅DM), with metabolic syndrome and diabetes mellitus (MS+DM), with neither metabolic syndrome nor diabetes mellitus (∅MS ∅DM), and with diabetes mellitus only (∅MS DM); *** p < 0.001.

**Figure 5:**
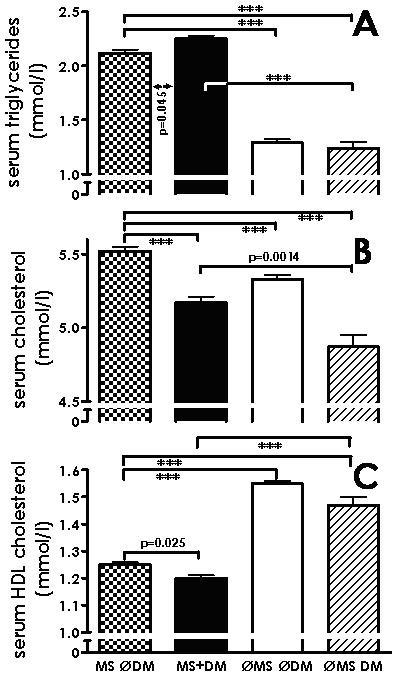
Serum lipids (mean ± SD; A: triglycerides, B: total cholesterol, C: HDL cholesterol) in patients with metabolic syndrome, but no diabetes mellitus (MS ∅DM), with metabolic syndrome and diabetes mellitus (MS+DM), with neither metabolic syndrome nor diabetes mellitus (∅MS ∅DM), and with diabetes mellitus only (∅MS DM); *** p < 0.001.

For significance levels and abbreviations see Figs. 1-5)

Similar findings were obtained with diastolic blood pressure (Figure 2, Table 1). Diastolic blood pressure was significantly higher in patients with metabolic syndrome alone, than in those with only diabetes mellitus. Moreover, patients with metabolic syndrome only showed a higher diastolic blood pressure than those with metabolic syndrome and diabetes mellitus.

The number of different antihypertensive therapies the patients were receiving is shown in Table 2. The percentage of patients receiving 1, 2 or ≥3 different treatments was similar in those with metabolic syndrome alone and in those with diabetes alone, with 34.4% and 38.8%, respectively, receiving ≥3 treatments. More patients (48.4%) with metabolic syndrome and diabetes, and fewer (28.0%) with neither metabolic syndrome nor diabetes were receiving ≥3 treatments.

**Table 2:** Number of antihypertensive therapies received by patients according to the presence or absence of metabolic syndrome and diabetes.

	**A**	**B**	**C**	**D**
**Number of different antihypertensive therapies**	**Metabolic syndrome but no diabetes ^a,b^** **(%)**	**Metabolic syndrome and diabetes ^b^** **(%)**	**No metabolic syndrome or diabetes ^c^** **(%)**	**No metabolic syndrome but diabetes** **(%)**

0	1.1	0.1	0.3	0.5

1	29.3	21.4	31.5	27.7

2	35.3	30.3	36.5	32.0

≥3	34.4	48.4	28.0	38.8

a: p < 0.01 vs. B; b: p < 0.01 vs. C; c: p < 0.05 vs. D. The other intergroup differences were not significant by the Chi square test

The next question to arise was which component of the metabolic syndrome, except blood glucose, may be associated with blood pressure. Figure 3 and Table 1 show body mass index (BMI) depending on the presence or absence of metabolic syndrome and diabetes mellitus. BMI was higher in patients with metabolic syndrome and diabetes than in those with metabolic syndrome alone. BMI in patients with metabolic syndrome only was significantly higher than in those with diabetes mellitus alone.

Waist circumference was highest in patients with metabolic syndrome whether or not they had concomitant diabetes mellitus. Patients with diabetes mellitus alone showed significantly lower waist circumference than those with metabolic syndrome alone (Figure 4, Table 1).

Next, we examined the lipid parameters obtained for their association with diabetes mellitus and the metabolic syndrome. Figure 5 and Table 1 show serum triglycerides, total and HDL cholesterol levels depending on the presence or absence of metabolic syndrome and diabetes mellitus. Serum triglycerides were highest in patients with metabolic syndrome with diabetes mellitus. Patients with metabolic syndrome only, had significantly higher serum triglycerides than patients with diabetes mellitus only (Figure 5A, Table 1). Similarly, serum cholesterol was highest in patients with metabolic syndrome only, whereas patients with diabetes mellitus only, had the lowest serum cholesterol (Figure 5B, Table 1). Serum HDL cholesterol levels showed a pattern largely inverse to that of serum triglycerides, with the lowest levels in patients with metabolic syndrome, either with or without diabetes mellitus, which were significantly lower than in patients with diabetes mellitus but no metabolic syndrome (Figure 5C, Table 1). Thus, the lipid parameters studied showed a similar pattern to those of systolic and diastolic blood pressure among the four groups of patients.

## Discussion

The findings show that blood pressure is influenced by the metabolic syndrome, irrespective of the presence or absence of diabetes mellitus. This finding was not a reflection of the antihypertensive treatments received by the patients, as those with metabolic syndrome alone and those with diabetes alone were receiving a similar number of treatments. Other factors may be more important for conferring resistance to antihypertensive treatment, since patients with diabetes mellitus alone, without accompanying metabolic syndrome, showed much lower blood pressure than patients with metabolic syndrome with or without diabetes mellitus. In addition, resistance to antihypertensive treatment may also reflect some doubts about target blood pressure values and low physician compliance in prescribing further therapy for patients with metabolic syndrome[1].

As disturbed glucose metabolism is present in both metabolic syndrome and diabetes mellitus, other factors of the metabolic syndrome are likely to be responsible for the tendency towards higher blood pressure in patients with metabolic syndrome. Indeed, when we evaluated the other components of the metabolic syndrome with respect to their impact on blood pressure, it was BMI and waist circumference which showed the closest association with blood pressure, both systolic and diastolic. On the other hand, the presence or absence of diabetes mellitus was not a major determinant of blood pressure. The above analyses were based on blood pressure values instead of blood pressure control rates. This seemed appropriate since blood pressure control rates were much lower in patients with diabetes mellitus than in patients with metabolic syndrome due to the fact that in patients with metabolic syndrome without diabetes mellitus the blood pressure target was < 140/90 mm Hg, but in patients with diabetes mellitus with or without metabolic syndrome the blood pressure target was < 130/80 mm Hg. Due to the different blood pressure targets, blood pressure control rates differed markedly depending on the presence or absence of diabetes mellitus.

These findings suggest that among the components of the metabolic syndrome, it is central obesity which affects blood pressure most. These findings are in line with the newer concepts on the pathophysiology of hypertension. The role of adipocytokines in the pathogenesis of hypertension has been of increasing interest in the last decade, and has markedly extended our view on the humoral mechanisms of hypertension [3-6]. Among the adipocytokines involved in the pathogenesis of hypertension, adiponectin and leptin have been most extensively studied. Recently, in a 5-year prospective study, serum adiponectin levels have been shown to be independent predictors of incident hypertension in a population normotensive at baseline [7].

Adiponectin, besides its effects on insulin sensitivity, may also act on the vasculature directly. Hypoadiponectinemia was found to be associated with an impaired endothelium-dependent vasodilation in humans and mice [8-10]. Conversely, adiponectin is a stimulator of nitric oxide (NO) production in endothelial cells [11,12]. In addition to adiponectin, other adipocytokines, such as leptin, have also been linked with the development of hypertension. Leptin increases peripheral sympathetic tone, and leptin-deficient mice show lower arterial blood pressure [13,14]. Among the many known adipocytokines, angiotensinogen has been found to be produced by adipocytes, and angiotensinogen can promote the development of hypertension by stimulating the production of angiotensin II [15,16].

There is also ample epidemiological evidence that obesity is one of the major causes of essential hypertension. Excess weight gain has been repeatedly shown to be one of the best predictors for the development of hypertension [17-19]. Moreover, from the Framingham Heart study it has been estimated that about 65% to 75% of the risk for hypertension can be attributed to excess weight [20].

Recent results from the National Health and Nutrition Examination Survey (NHANES) support the eminent role of obesity in the development of hypertension [21]: between NHANES II (1988-1994) and NHANES 1999-2004, the age-standardized hypertension prevalence increased from 24.4% to 28.9%. Depending on gender and ethnicity, between 20% and 80% of this increase could be attributed to increasing BMI. Although the authors did not report control rates depending on BMI, blood pressure distribution within the population was shifted towards higher values with increasing BMI.

The relative contributions of subcutaneous and visceral fat to the development of hypertension have been elucidated in further studies. It is visceral fat which has been identified as the more prominent factor inducing hypertension [22]: quantification of adipose tissue in different locations by computer tomography revealed that, in a group of 300 Japanese Americans, the amount of visceral fat, but not subcutaneous fat, conferred a risk of incident hypertension during the follow-up of up to 11 years.

Blood pressure is also associated with humoral components of the metabolic syndrome. Low HDL levels are known to confer an increased cardiovascular risk. Moreover, a direct association between low serum HDL levels and blood pressure has been shown in elderly hypertensive patients [23]. The mechanistic background of this association may be that HDL contains important lipid mediators such as sphingosine-1-phosphate, sphingosylphosphorylcholine, and lysosulphatide which induce NO-dependent vasorelaxation via the lysophospholipid receptor S1P3 [24]. Thereby HDL restores endothelial function by improving NO availability and hence favours vasorelaxation.

As in the present survey we studied patients receiving antihypertensive treatment, the results do not imply a higher prevalence of hypertension with increasing BMI, but show less effective blood pressure control in obese patients. This finding infers that in obesity-related hypertension other pathogenetic mechanisms may be involved than occur in other forms of essential hypertension.

The data in this survey show that metabolic syndrome is not only associated with higher blood pressure but also with the poor response to treatment. Interestingly, among the components of the metabolic syndrome, it is not impaired glucose tolerance which is associated with the response to antihypertensive treatment. Instead, both visceral obesity and the dyslipidemia of the metabolic syndrome, i.e. hypertriglyceridemia and low HDL cholesterol levels, are associated with resistance to antihypertensive treatment.

There have been few studies addressing the relative contributions of single components of the metabolic syndrome to the development of hypertension and vascular disease [25]. The GOOD registry offers an appropriate database to answer these questions. Earlier publications of the GOOD data focused on the control of blood pressure depending on the co-occurrence of the metabolic syndrome [1] and on regional differences in blood pressure control [2]. The DIG study addressed a similar question as the present study; Hanefeld et al. [26] studied the relative contributions of the different components of the metabolic syndrome to the development of atherosclerotic vascular disease. In contrast to the present study, the DIG study did not assess the impact of the components of the metabolic syndrome on the development of hypertension. Similarly to the present study, diabetes mellitus alone did not contribute significantly to the development of atherosclerotic vascular disease, whereas the combination of diabetes mellitus with hypertension and dyslipidemia was closely associated with vascular disease.

## Conclusion

Among the components of the metabolic syndrome, impaired glucose tolerance is not associated with the poor response to antihypertensive treatment. Instead, other components of the metabolic syndrome, viz. visceral obesity and dyslipidemia, hypertriglyceridemia and low HDL cholesterol levels, are associated with resistance to antihypertensive treatment.

## Competing interests

Dr Naditch-Brûlé declares to be an employee of Sanofi-Aventis. All other authors have attended advisory boards and have held lectures for a number of pharmaceutical companies including the sponsor.

## Authors' contributions

WZ prepared the manuscript, LN-B provided statistical expertise, SP created the database, CF developed the hypotheses tested, SEK significantly contributed to the design of the GOOD trial. All authors read and approved the final manuscript.
